# Alcohol assessment and feedback by e-mail for university students: main findings from the AMADEUS-1 randomised controlled trial

**DOI:** 10.1186/1940-0640-8-S1-A48

**Published:** 2013-09-04

**Authors:** Jim McCambridge, Marcus Bendtsen, Nadine Karlsson, Ian R White, Per Nilsen, Preben Bendtsen

**Affiliations:** 1 Faculty of Public Health & Policy, London School of Hygiene & Tropical Medicine, UK; 2 Department of Medicine and Health, Linköping University, Sweden; 3 Department of Computer and Information Science, Linköping University, Sweden; 4 MRC Biostatistics Unit, Institute of Public Health, Cambridge University, UK

## Background

Brief interventions can be efficacious in changing alcohol consumption and related problems and increasingly take advantage of the internet to reach high risk populations such as students.

## Aims

To evaluate the effectiveness of a brief online intervention, part of the national strategic response in Sweden, controlling for the possible effects of the research process.

## Methods

A three arm parallel groups design permitted exploration of the magnitude of the feedback and assessment component effects via randomisation to fully automated: 1) routine practice assessment and feedback; 2) assessment only without feedback; or 3) no contact and thus neither assessment nor feedback. The study was undertaken simultaneously in two universities randomizing the e-mail addresses of all 14,910 students (4,969, 4969 and 4972 respectively to Groups 1-3) who were entirely blinded to trial participation. Outcomes were evaluated after 3 months via an invitation to participate in a brief cross-sectional lifestyle survey.

## Results

Overall, 52% (n=7,809) of all students completed follow-up, with small differences in attrition between the three groups (2,546, 2,594 and 2,669 respectively in Groups 1-3). For each of the two primary outcomes, there was one statistically significant difference between groups, with Group 1 having 3.7% fewer risky drinkers at follow-up than Group 3 (P=0.006) and Group 2 scoring 0.16 points lower than Group 3 on the AUDIT-C (P=0.039).

## Conclusions

This study provides some evidence of population-level benefit attained through intervening with individual students.

## Trial registration

ISRCTN28328154

